# An Erroneous Diagnosis

**Published:** 1898-04-23

**Authors:** 


### AN ERRONEOUS DIAGNOSIS.

A case reported in the *British Medical Journal Epitome* shows how easy it is, unless a full examination be made, to fall into error in the diagnosis of even the simplest conditions. In this case a young woman, aged 23, a primipara, was considered to be three months pregnant, having previously to this, her first, pregnancy been quite regular. For a few days she had suffered from pain on defæcation, and for three days there had been complete obstruction, neither flatus nor fæces passing, in spite of purgatives and enemata. The pulse was good and the abdomen moderately distended with flatus, and not extremely tender. The cervix lay forwards and to the left, while in the posterior fornix a swelling of the size of the uterus at the third month could be detected. The case was looked upon as one of retroversion of the pregnant uterus, and under chloroform the uterus was apparently replaced with ease. The after-history, however, showed that this had been a case of ectopic pregnancy. What probably happened at the time of the replacement was that the sac was ruptured; and, although the patient got well, it is clear that she ran a considerable risk. The symptoms, both general and vaginal, were such as to lead to error, but no doubt a complete bimanual examination would have cleared up the condition of affairs.

